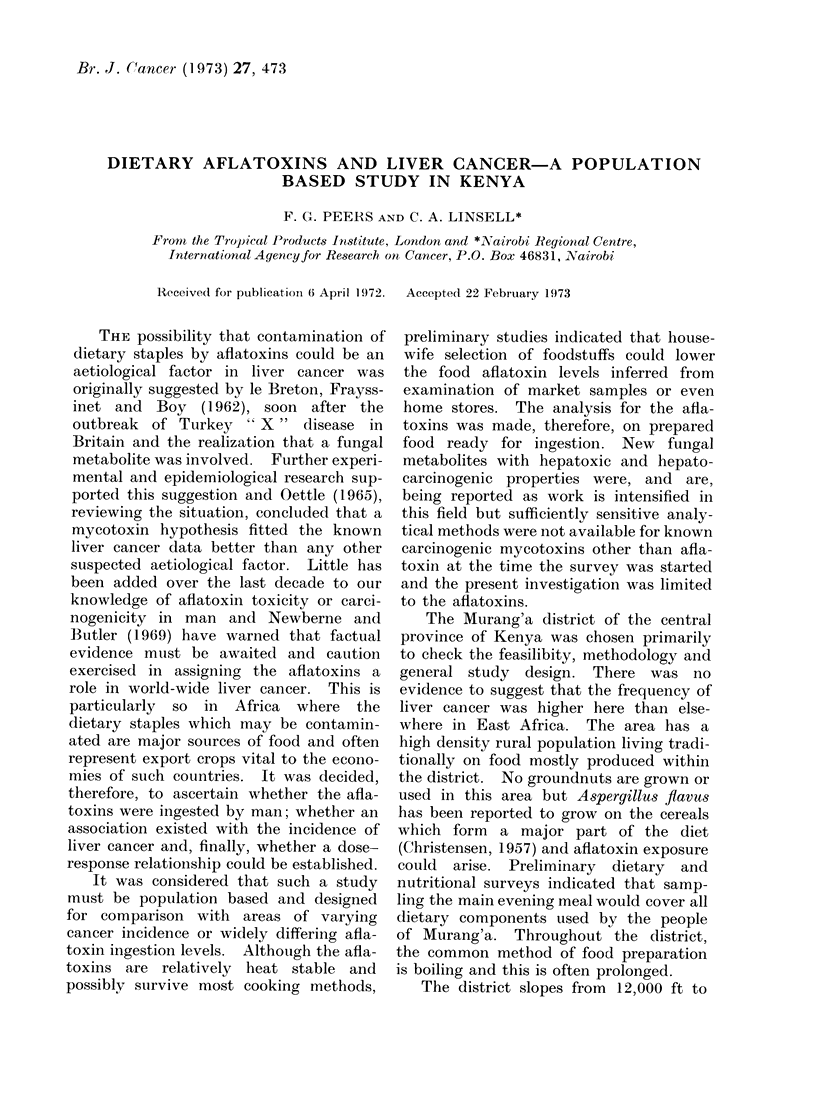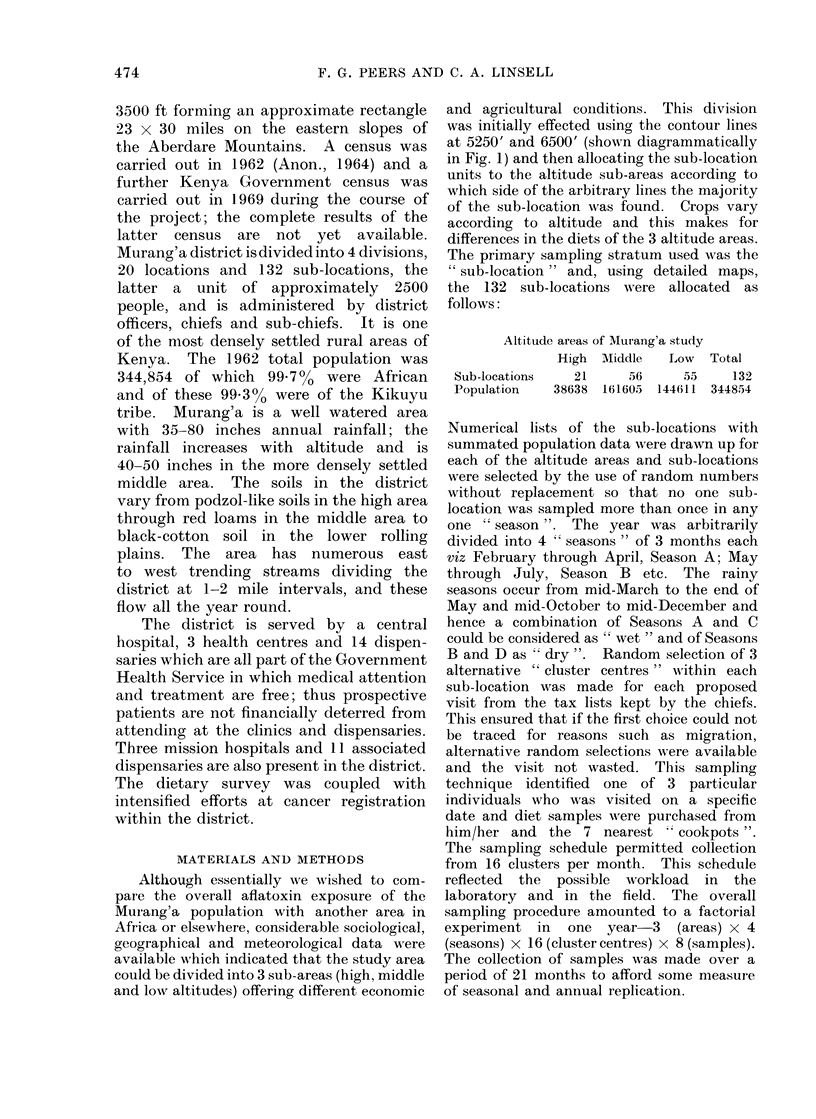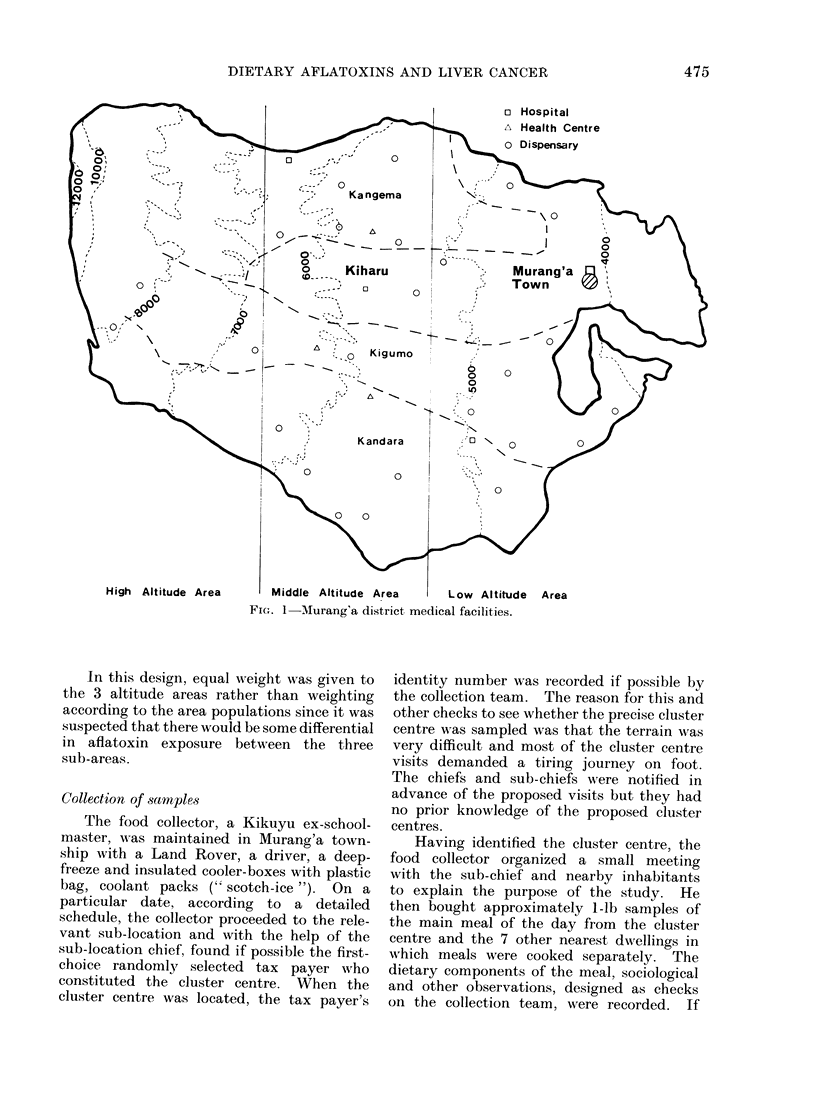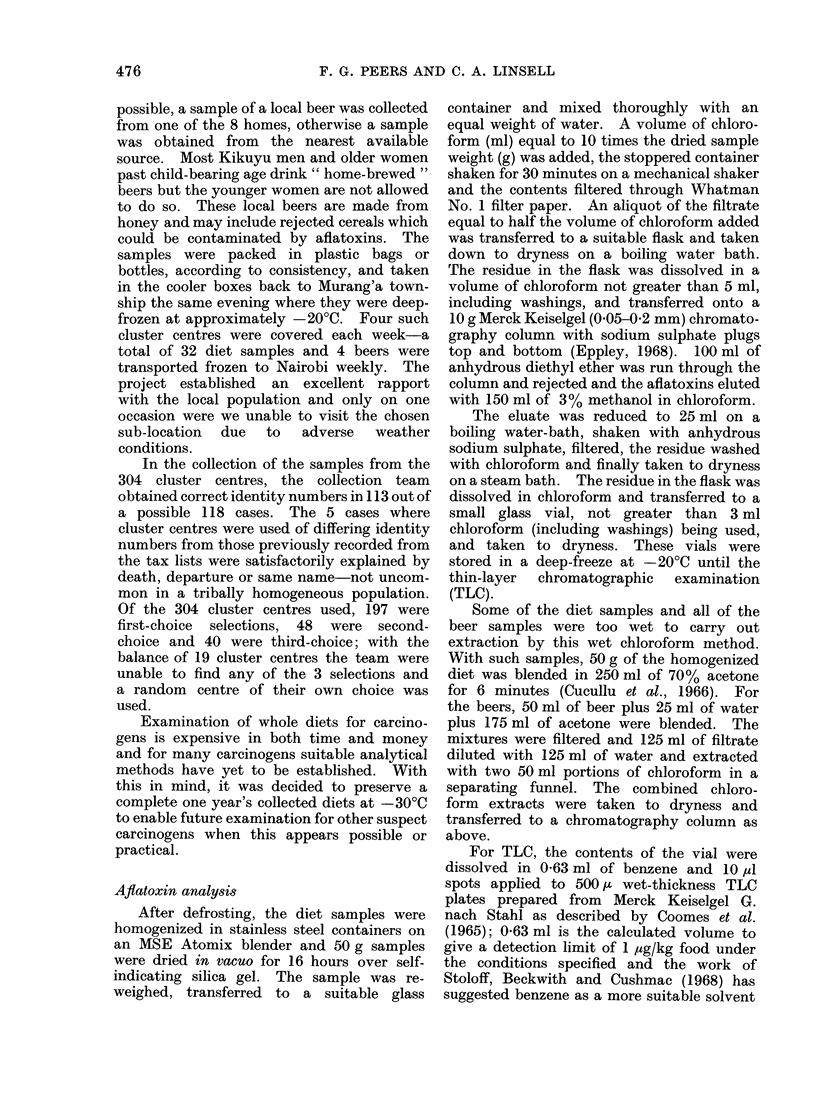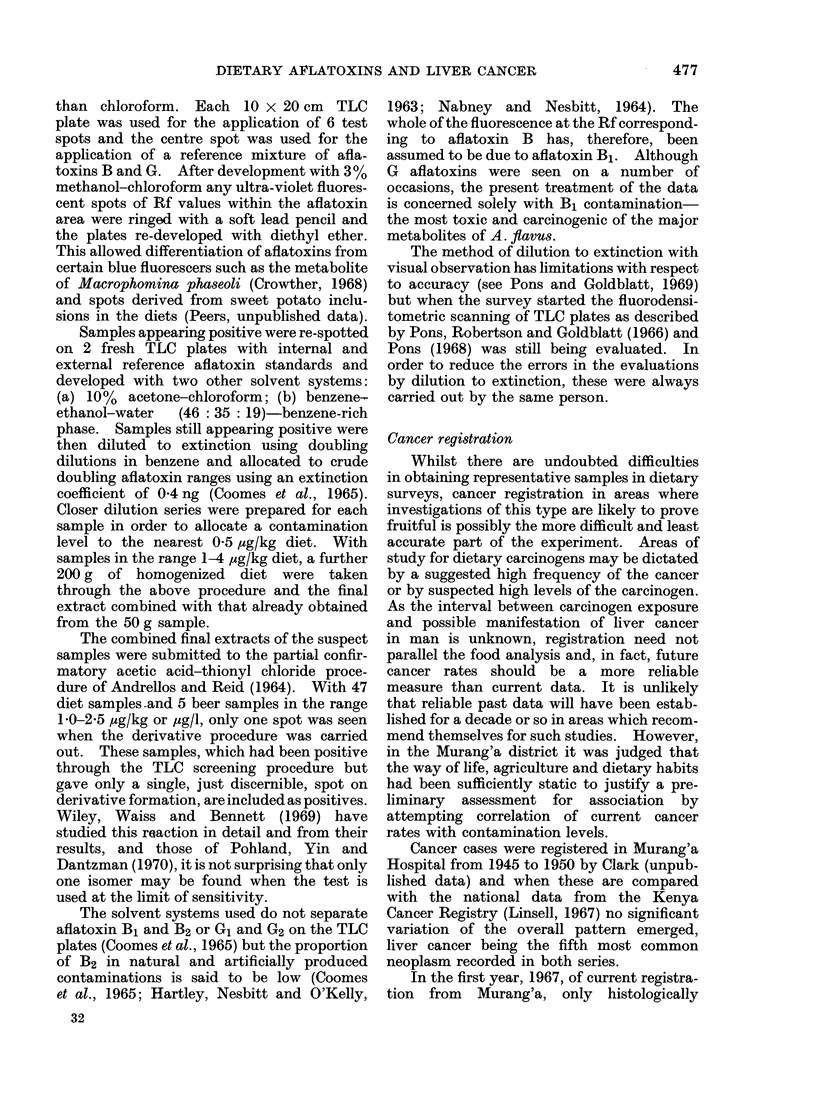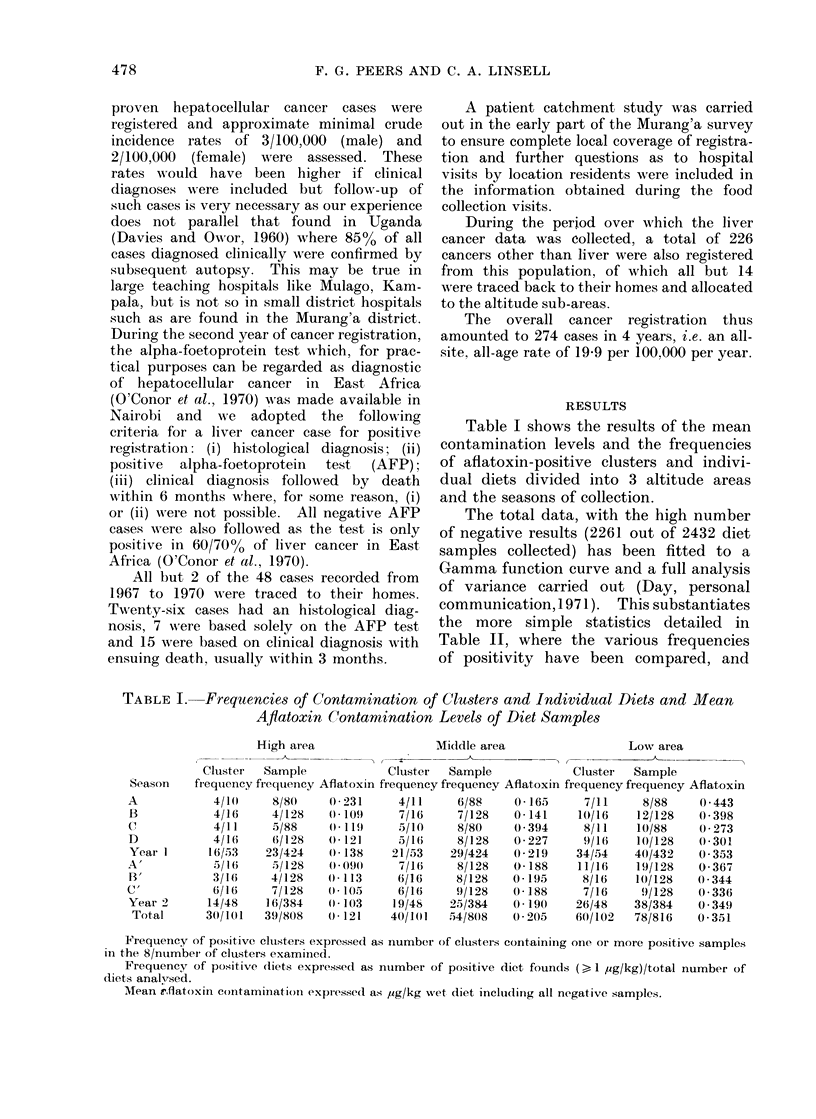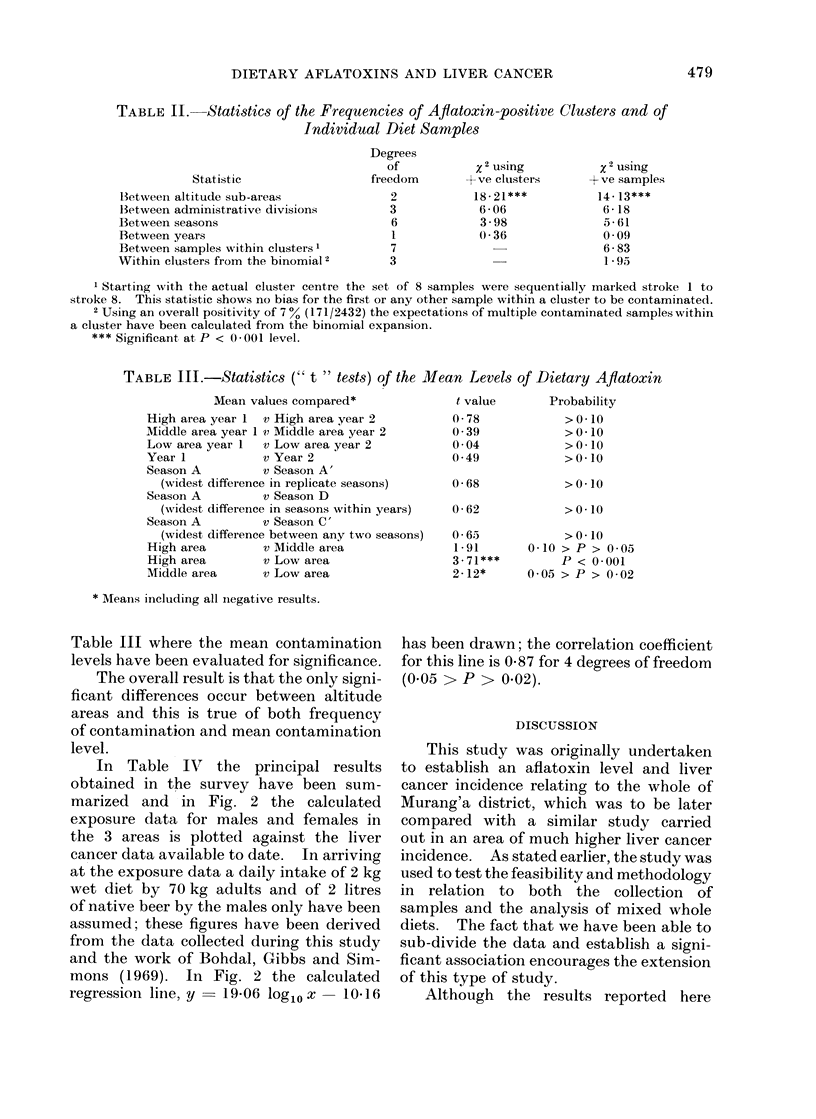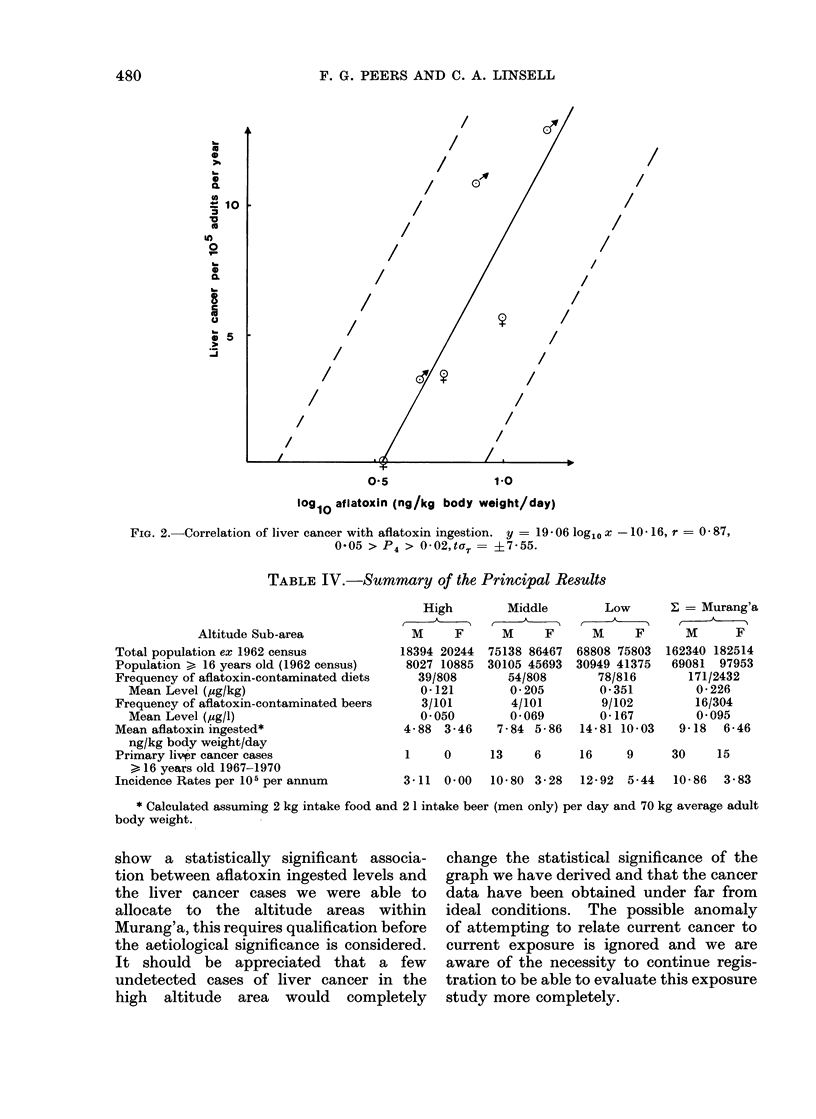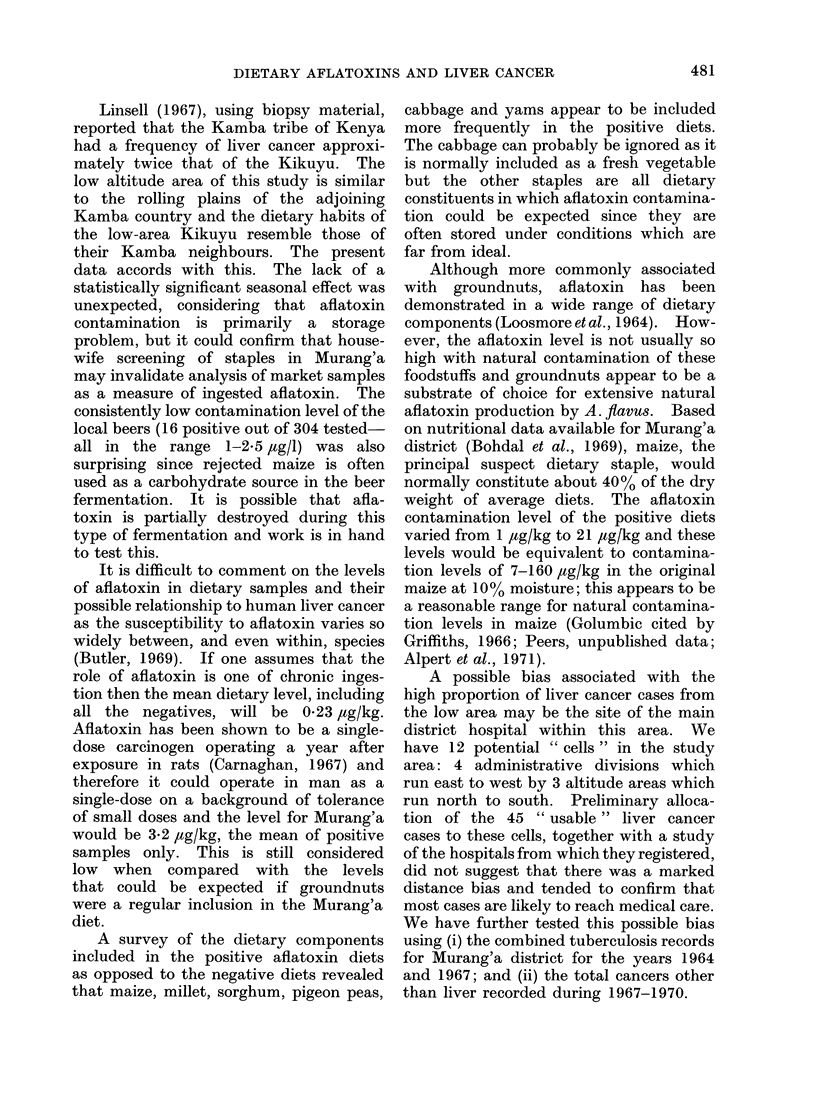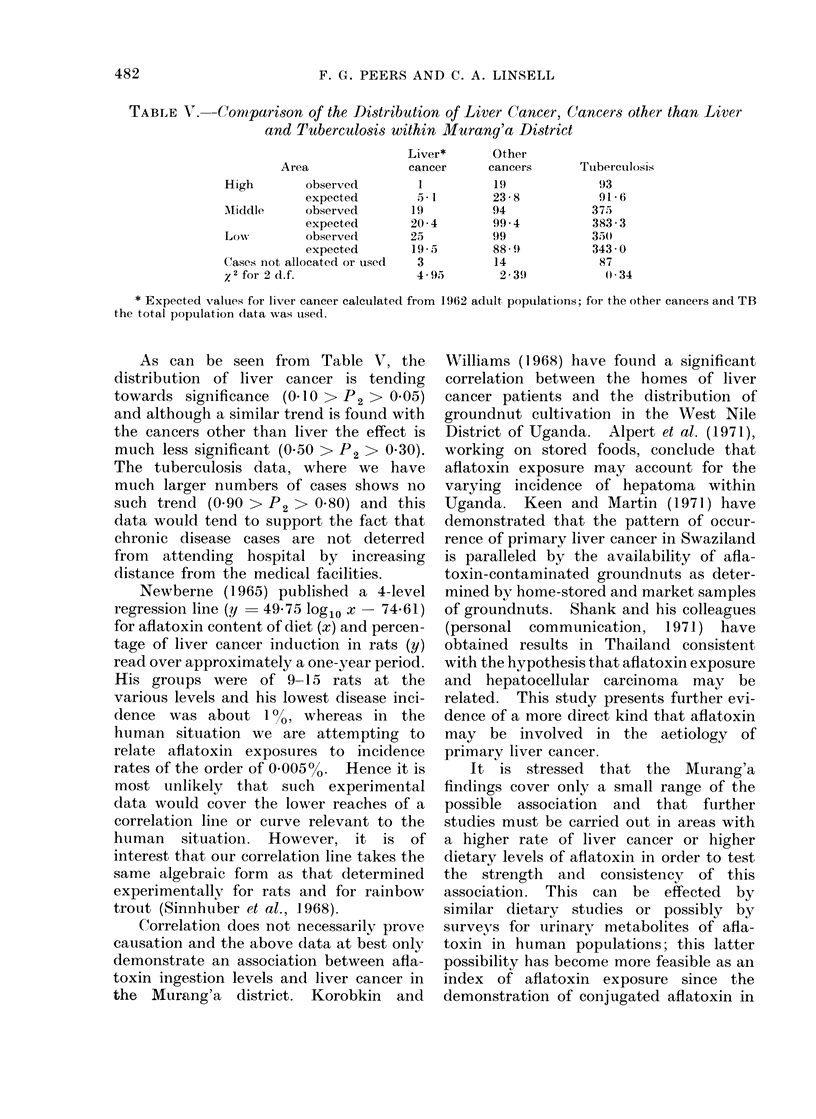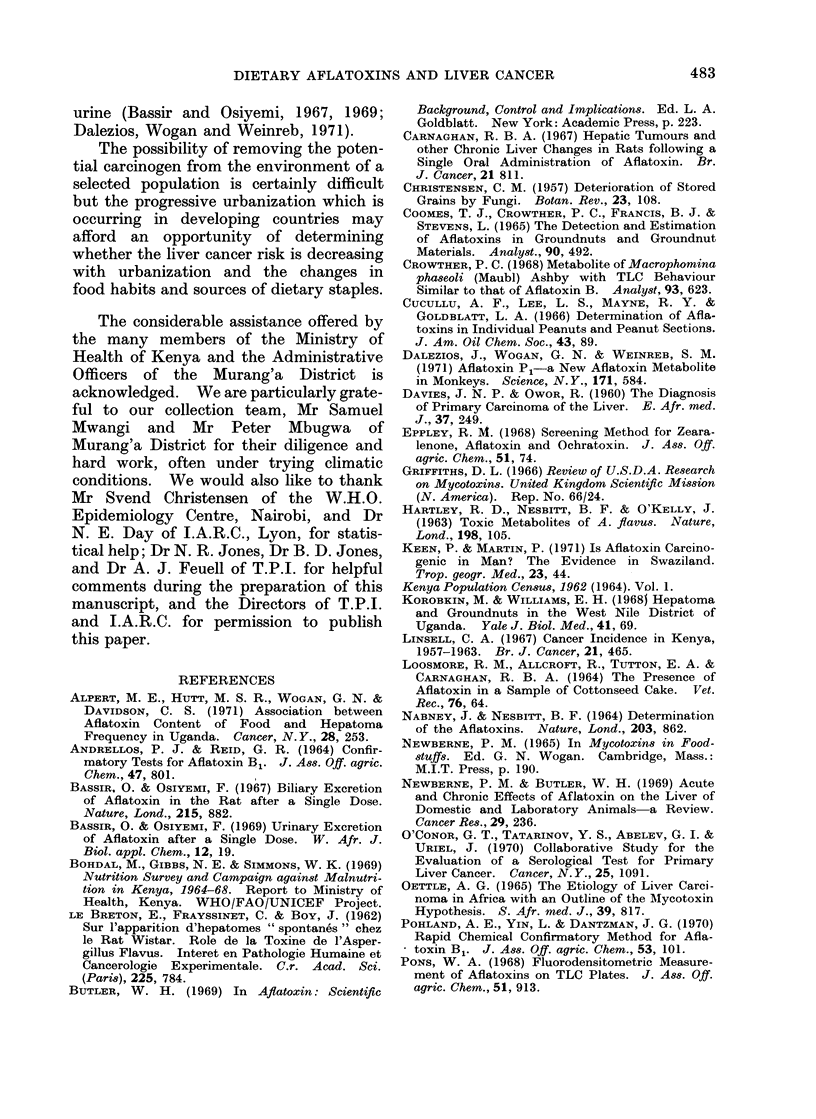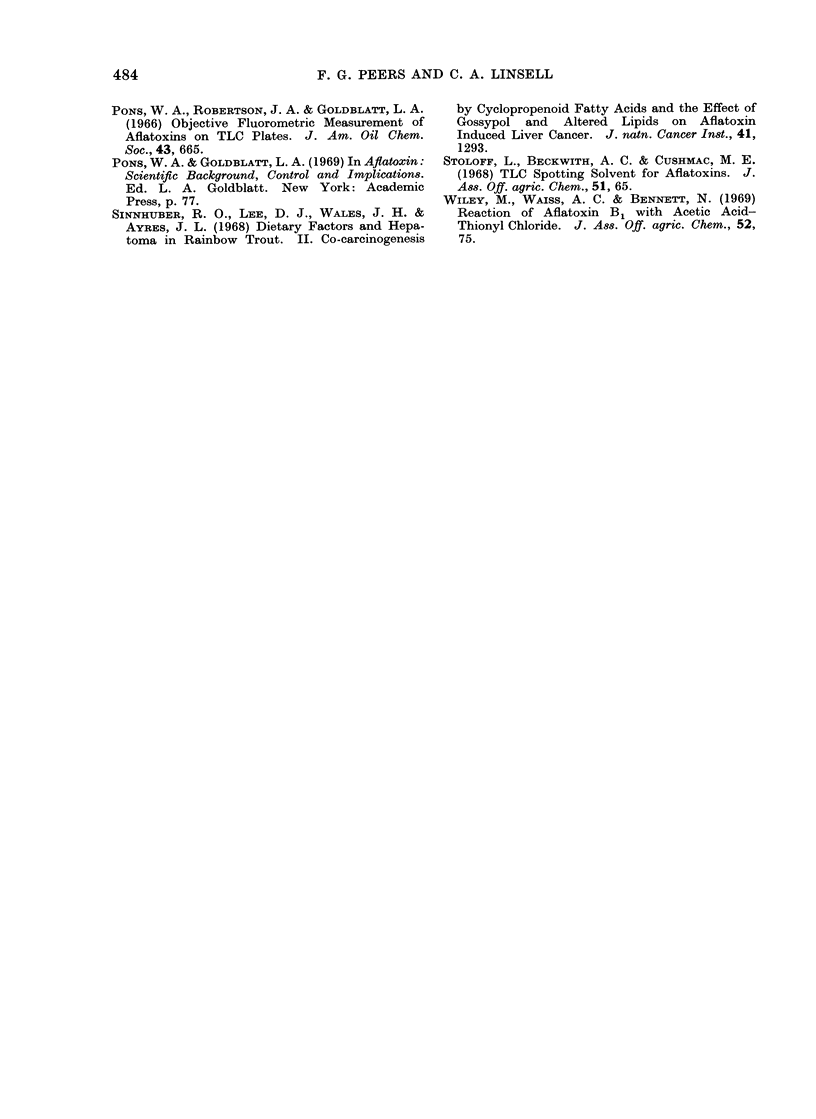# Dietary aflatoxins and liver cancer--a population based study in Kenya.

**DOI:** 10.1038/bjc.1973.60

**Published:** 1973-06

**Authors:** F. G. Peers, C. A. Linsell


*Br. J. Cancer* (1973) **27**, 473

### DIETARY AFLATOXINS AND LIVER CANCER—A POPULATION BASED STUDY IN KENYA

F. G. PEERS AND C. A. LINSELL*

*From the Tropical Products Institute, London and *Nairobi Regional Centre, International Agency for Research on Cancer, P.O. Box 46831, Nairobi*

Received for publication 6 April 1972. Accepted 22 February 1973

THE possibility that contamination of dietary staples by aflatoxins could be an aetiological factor in liver cancer was originally suggested by le Breton, Frayssinet and Boy (1962), soon after the outbreak of Turkey " X " disease in Britain and the realization that a fungal metabolite was involved. Further experimental and epidemiological research supported this suggestion and Oettle (1965), reviewing the situation, concluded that a mycotoxin hypothesis fitted the known liver cancer data better than any other suspected aetiological factor. Little has been added over the last decade to our knowledge of aflatoxin toxicity or carcinogenicity in man and Newberne and Butler (1969) have warned that factual evidence must be awaited and caution exercised in assigning the aflatoxins a role in world-wide liver cancer. This is particularly so in Africa where the dietary staples which may be contaminated are major sources of food and often represent export crops vital to the economies of such countries. It was decided, therefore, to ascertain whether the aflatoxins were ingested by man; whether an association existed with the incidence of liver cancer and, finally, whether a dose–response relationship could be established.

It was considered that such a study must be population based and designed for comparison with areas of varying cancer incidence or widely differing aflatoxin ingestion levels. Although the aflatoxins are relatively heat stable and possibly survive most cooking methods, preliminary studies indicated that housewife selection of foodstuffs could lower the food aflatoxin levels inferred from examination of market samples or even home stores. The analysis for the aflatoxins was made, therefore, on prepared food ready for ingestion. New fungal metabolites with hepatoxic and hepatocarcinogenic properties were, and are, being reported as work is intensified in this field but sufficiently sensitive analytical methods were not available for known carcinogenic mycotoxins other than aflatoxin at the time the survey was started and the present investigation was limited to the aflatoxins.

The Murang'a district of the central province of Kenya was chosen primarily to check the feasilibity, methodology and general study design. There was no evidence to suggest that the frequency of liver cancer was higher here than elsewhere in East Africa. The area has a high density rural population living traditionally on food mostly produced within the district. No groundnuts are grown or used in this area but *Aspergillus flavus* has been reported to grow on the cereals which form a major part of the diet (Christensen, 1957) and aflatoxin exposure could arise. Preliminary dietary and nutritional surveys indicated that sampling the main evening meal would cover all dietary components used by the people of Murang'a. Throughout the district, the common method of food preparation is boiling and this is often prolonged.

The district slopes from 12,000 ft to

3500 ft forming an approximate rectangle 23 × 30 miles on the eastern slopes of the Aberdare Mountains. A census was carried out in 1962 (Anon., 1964) and a further Kenya Government census was carried out in 1969 during the course of the project; the complete results of the latter census are not yet available. Murang'a district is divided into 4 divisions, 20 locations and 132 sub-locations, the latter a unit of approximately 2500 people, and is administered by district officers, chiefs and sub-chiefs. It is one of the most densely settled rural areas of Kenya. The 1962 total population was 344,854 of which 99·7% were African and of these 99·3% were of the Kikuyu tribe. Murang'a is a well watered area with 35–80 inches annual rainfall; the rainfall increases with altitude and is 40–50 inches in the more densely settled middle area. The soils in the district vary from podzol-like soils in the high area through red loams in the middle area to black-cotton soil in the lower rolling plains. The area has numerous east to west trending streams dividing the district at 1–2 mile intervals, and these flow all the year round.

The district is served by a central hospital, 3 health centres and 14 dispensaries which are all part of the Government Health Service in which medical attention and treatment are free; thus prospective patients are not financially deterred from attending at the clinics and dispensaries. Three mission hospitals and 11 associated dispensaries are also present in the district. The dietary survey was coupled with intensified efforts at cancer registration within the district.

### MATERIALS AND METHODS

Although essentially we wished to compare the overall aflatoxin exposure of the Murang'a population with another area in Africa or elsewhere, considerable sociological, geographical and meteorological data were available which indicated that the study area could be divided into 3 sub-areas (high, middle and low altitudes) offering different economic and agricultural conditions. This division was initially effected using the contour lines at 5250′ and 6500′ (shown diagrammatically in Fig. 1) and then allocating the sub-location units to the altitude sub-areas according to which side of the arbitrary lines the majority of the sub-location was found. Crops vary according to altitude and this makes for differences in the diets of the 3 altitude areas. The primary sampling stratum used was the " sub-location " and, using detailed maps, the 132 sub-locations were allocated as follows:

Altitude areas of Murang'a study

| | High | Middle | Low | Total |
|---|---|---|---|---|
| Sub-locations | 21 | 56 | 55 | 132 |
| Population | 38638 | 161605 | 144611 | 344854 |

Numerical lists of the sub-locations with summated population data were drawn up for each of the altitude areas and sub-locations were selected by the use of random numbers without replacement so that no one sub-location was sampled more than once in any one " season ". The year was arbitrarily divided into 4 " seasons " of 3 months each *viz* February through April, Season A; May through July, Season B etc. The rainy seasons occur from mid-March to the end of May and mid-October to mid-December and hence a combination of Seasons A and C could be considered as " wet " and of Seasons B and D as " dry ". Random selection of 3 alternative " cluster centres " within each sub-location was made for each proposed visit from the tax lists kept by the chiefs. This ensured that if the first choice could not be traced for reasons such as migration, alternative random selections were available and the visit not wasted. This sampling technique identified one of 3 particular individuals who was visited on a specific date and diet samples were purchased from him/her and the 7 nearest " cookpots ". The sampling schedule permitted collection from 16 clusters per month. This schedule reflected the possible workload in the laboratory and in the field. The overall sampling procedure amounted to a factorial experiment in one year—3 (areas) × 4 (seasons) × 16 (cluster centres) × 8 (samples). The collection of samples was made over a period of 21 months to afford some measure of seasonal and annual replication.

FIG. 1—Murang'a district medical facilities.

In this design, equal weight was given to the 3 altitude areas rather than weighting according to the area populations since it was suspected that there would be some differential in aflatoxin exposure between the three sub-areas.

*Collection of samples*

The food collector, a Kikuyu ex-schoolmaster, was maintained in Murang'a township with a Land Rover, a driver, a deep-freeze and insulated cooler-boxes with plastic bag, coolant packs ("scotch-ice"). On a particular date, according to a detailed schedule, the collector proceeded to the relevant sub-location and with the help of the sub-location chief, found if possible the first-choice randomly selected tax payer who constituted the cluster centre. When the cluster centre was located, the tax payer's identity number was recorded if possible by the collection team. The reason for this and other checks to see whether the precise cluster centre was sampled was that the terrain was very difficult and most of the cluster centre visits demanded a tiring journey on foot. The chiefs and sub-chiefs were notified in advance of the proposed visits but they had no prior knowledge of the proposed cluster centres.

Having identified the cluster centre, the food collector organized a small meeting with the sub-chief and nearby inhabitants to explain the purpose of the study. He then bought approximately 1-lb samples of the main meal of the day from the cluster centre and the 7 other nearest dwellings in which meals were cooked separately. The dietary components of the meal, sociological and other observations, designed as checks on the collection team, were recorded. If possible, a sample of a local beer was collected from one of the 8 homes, otherwise a sample was obtained from the nearest available source. Most Kikuyu men and older women past child-bearing age drink " home-brewed " beers but the younger women are not allowed to do so. These local beers are made from honey and may include rejected cereals which could be contaminated by aflatoxins. The samples were packed in plastic bags or bottles, according to consistency, and taken in the cooler boxes back to Murang'a township the same evening where they were deep-frozen at approximately −20°C. Four such cluster centres were covered each week—a total of 32 diet samples and 4 beers were transported frozen to Nairobi weekly. The project established an excellent rapport with the local population and only on one occasion were we unable to visit the chosen sub-location due to adverse weather conditions.

In the collection of the samples from the 304 cluster centres, the collection team obtained correct identity numbers in 113 out of a possible 118 cases. The 5 cases where cluster centres were used of differing identity numbers from those previously recorded from the tax lists were satisfactorily explained by death, departure or same name—not uncommon in a tribally homogeneous population. Of the 304 cluster centres used, 197 were first-choice selections, 48 were second-choice and 40 were third-choice; with the balance of 19 cluster centres the team were unable to find any of the 3 selections and a random centre of their own choice was used.

Examination of whole diets for carcinogens is expensive in both time and money and for many carcinogens suitable analytical methods have yet to be established. With this in mind, it was decided to preserve a complete one year's collected diets at −30°C to enable future examination for other suspect carcinogens when this appears possible or practical.

*Aflatoxin analysis*

After defrosting, the diet samples were homogenized in stainless steel containers on an MSE Atomix blender and 50 g samples were dried *in vacuo* for 16 hours over self-indicating silica gel. The sample was re-weighed, transferred to a suitable glass container and mixed thoroughly with an equal weight of water. A volume of chloroform (ml) equal to 10 times the dried sample weight (g) was added, the stoppered container shaken for 30 minutes on a mechanical shaker and the contents filtered through Whatman No. 1 filter paper. An aliquot of the filtrate equal to half the volume of chloroform added was transferred to a suitable flask and taken down to dryness on a boiling water bath. The residue in the flask was dissolved in a volume of chloroform not greater than 5 ml, including washings, and transferred onto a 10 g Merck Keiselgel (0·05–0·2 mm) chromatography column with sodium sulphate plugs top and bottom (Eppley, 1968). 100 ml of anhydrous diethyl ether was run through the column and rejected and the aflatoxins eluted with 150 ml of 3% methanol in chloroform.

The eluate was reduced to 25 ml on a boiling water-bath, shaken with anhydrous sodium sulphate, filtered, the residue washed with chloroform and finally taken to dryness on a steam bath. The residue in the flask was dissolved in chloroform and transferred to a small glass vial, not greater than 3 ml chloroform (including washings) being used, and taken to dryness. These vials were stored in a deep-freeze at −20°C until the thin-layer chromatographic examination (TLC).

Some of the diet samples and all of the beer samples were too wet to carry out extraction by this wet chloroform method. With such samples, 50 g of the homogenized diet was blended in 250 ml of 70% acetone for 6 minutes (Cucullu *et al.*, 1966). For the beers, 50 ml of beer plus 25 ml of water plus 175 ml of acetone were blended. The mixtures were filtered and 125 ml of filtrate diluted with 125 ml of water and extracted with two 50 ml portions of chloroform in a separating funnel. The combined chloroform extracts were taken to dryness and transferred to a chromatography column as above.

For TLC, the contents of the vial were dissolved in 0·63 ml of benzene and 10 $\mu l$ spots applied to 500 $\mu$ wet-thickness TLC plates prepared from Merck Keiselgel G. nach Stahl as described by Coomes *et al.* (1965); 0·63 ml is the calculated volume to give a detection limit of 1 $\mu$g/kg food under the conditions specified and the work of Stoloff, Beckwith and Cushmac (1968) has suggested benzene as a more suitable solvent than chloroform. Each 10 × 20 cm TLC plate was used for the application of 6 test spots and the centre spot was used for the application of a reference mixture of aflatoxins B and G. After development with 3% methanol–chloroform any ultra-violet fluorescent spots of Rf values within the aflatoxin area were ringed with a soft lead pencil and the plates re-developed with diethyl ether. This allowed differentiation of aflatoxins from certain blue fluorescers such as the metabolite of *Macrophomina phaseoli* (Crowther, 1968) and spots derived from sweet potato inclusions in the diets (Peers, unpublished data).

Samples appearing positive were re-spotted on 2 fresh TLC plates with internal and external reference aflatoxin standards and developed with two other solvent systems: (a) 10% acetone–chloroform; (b) benzene–ethanol–water (46 : 35 : 19)—benzene-rich phase. Samples still appearing positive were then diluted to extinction using doubling dilutions in benzene and allocated to crude doubling aflatoxin ranges using an extinction coefficient of 0·4 ng (Coomes *et al.*, 1965). Closer dilution series were prepared for each sample in order to allocate a contamination level to the nearest 0·5 μg/kg diet. With samples in the range 1–4 μg/kg diet, a further 200 g of homogenized diet were taken through the above procedure and the final extract combined with that already obtained from the 50 g sample.

The combined final extracts of the suspect samples were submitted to the partial confirmatory acetic acid–thionyl chloride procedure of Andrellos and Reid (1964). With 47 diet samples and 5 beer samples in the range 1·0–2·5 μg/kg or μg/l, only one spot was seen when the derivative procedure was carried out. These samples, which had been positive through the TLC screening procedure but gave only a single, just discernible, spot on derivative formation, are included as positives. Wiley, Waiss and Bennett (1969) have studied this reaction in detail and from their results, and those of Pohland, Yin and Dantzman (1970), it is not surprising that only one isomer may be found when the test is used at the limit of sensitivity.

The solvent systems used do not separate aflatoxin $B_1$ and $B_2$ or $G_1$ and $G_2$ on the TLC plates (Coomes *et al.*, 1965) but the proportion of $B_2$ in natural and artificially produced contaminations is said to be low (Coomes *et al.*, 1965; Hartley, Nesbitt and O'Kelly, 1963; Nabney and Nesbitt, 1964). The whole of the fluorescence at the Rf corresponding to aflatoxin B has, therefore, been assumed to be due to aflatoxin $B_1$. Although G aflatoxins were seen on a number of occasions, the present treatment of the data is concerned solely with $B_1$ contamination—the most toxic and carcinogenic of the major metabolites of *A. flavus*.

The method of dilution to extinction with visual observation has limitations with respect to accuracy (see Pons and Goldblatt, 1969) but when the survey started the fluorodensitometric scanning of TLC plates as described by Pons, Robertson and Goldblatt (1966) and Pons (1968) was still being evaluated. In order to reduce the errors in the evaluations by dilution to extinction, these were always carried out by the same person.

*Cancer registration*

Whilst there are undoubted difficulties in obtaining representative samples in dietary surveys, cancer registration in areas where investigations of this type are likely to prove fruitful is possibly the more difficult and least accurate part of the experiment. Areas of study for dietary carcinogens may be dictated by a suggested high frequency of the cancer or by suspected high levels of the carcinogen. As the interval between carcinogen exposure and possible manifestation of liver cancer in man is unknown, registration need not parallel the food analysis and, in fact, future cancer rates should be a more reliable measure than current data. It is unlikely that reliable past data will have been established for a decade or so in areas which recommend themselves for such studies. However, in the Murang'a district it was judged that the way of life, agriculture and dietary habits had been sufficiently static to justify a preliminary assessment for association by attempting correlation of current cancer rates with contamination levels.

Cancer cases were registered in Murang'a Hospital from 1945 to 1950 by Clark (unpublished data) and when these are compared with the national data from the Kenya Cancer Registry (Linsell, 1967) no significant variation of the overall pattern emerged, liver cancer being the fifth most common neoplasm recorded in both series.

In the first year, 1967, of current registration from Murang'a, only histologically proven hepatocellular cancer cases were registered and approximate minimal crude incidence rates of 3/100,000 (male) and 2/100,000 (female) were assessed. These rates would have been higher if clinical diagnoses were included but follow-up of such cases is very necessary as our experience does not parallel that found in Uganda (Davies and Owor, 1960) where 85% of all cases diagnosed clinically were confirmed by subsequent autopsy. This may be true in large teaching hospitals like Mulago, Kampala, but is not so in small district hospitals such as are found in the Murang'a district. During the second year of cancer registration, the alpha-foetoprotein test which, for practical purposes can be regarded as diagnostic of hepatocellular cancer in East Africa (O'Conor *et al.*, 1970) was made available in Nairobi and we adopted the following criteria for a liver cancer case for positive registration: (i) histological diagnosis; (ii) positive alpha-foetoprotein test (AFP); (iii) clinical diagnosis followed by death within 6 months where, for some reason, (i) or (ii) were not possible. All negative AFP cases were also followed as the test is only positive in 60/70% of liver cancer in East Africa (O'Conor *et al.*, 1970).

All but 2 of the 48 cases recorded from 1967 to 1970 were traced to their homes. Twenty-six cases had an histological diagnosis, 7 were based solely on the AFP test and 15 were based on clinical diagnosis with ensuing death, usually within 3 months.

A patient catchment study was carried out in the early part of the Murang'a survey to ensure complete local coverage of registration and further questions as to hospital visits by location residents were included in the information obtained during the food collection visits.

During the period over which the liver cancer data was collected, a total of 226 cancers other than liver were also registered from this population, of which all but 14 were traced back to their homes and allocated to the altitude sub-areas.

The overall cancer registration thus amounted to 274 cases in 4 years, *i.e.* an all-site, all-age rate of 19·9 per 100,000 per year.

### RESULTS

Table I shows the results of the mean contamination levels and the frequencies of aflatoxin-positive clusters and individual diets divided into 3 altitude areas and the seasons of collection.

The total data, with the high number of negative results (2261 out of 2432 diet samples collected) has been fitted to a Gamma function curve and a full analysis of variance carried out (Day, personal communication, 1971). This substantiates the more simple statistics detailed in Table II, where the various frequencies of positivity have been compared, and

TABLE I.—*Frequencies of Contamination of Clusters and Individual Diets and Mean Aflatoxin Contamination Levels of Diet Samples*

| | High area | | | Middle area | | | Low area | | |
|---|---|---|---|---|---|---|---|---|---|
| Season | Cluster frequency | Sample frequency | Aflatoxin | Cluster frequency | Sample frequency | Aflatoxin | Cluster frequency | Sample frequency | Aflatoxin |
| A | 4/10 | 8/80 | 0·231 | 4/11 | 6/88 | 0·165 | 7/11 | 8/88 | 0·443 |
| B | 4/16 | 4/128 | 0·109 | 7/16 | 7/128 | 0·141 | 10/16 | 12/128 | 0·398 |
| C | 4/11 | 5/88 | 0·119 | 5/10 | 8/80 | 0·394 | 8/11 | 10/88 | 0·273 |
| D | 4/16 | 6/128 | 0·121 | 5/16 | 8/128 | 0·227 | 9/16 | 10/128 | 0·301 |
| Year 1 | 16/53 | 23/424 | 0·138 | 21/53 | 29/424 | 0·219 | 34/54 | 40/432 | 0·353 |
| A′ | 5/16 | 5/128 | 0·090 | 7/16 | 8/128 | 0·188 | 11/16 | 19/128 | 0·367 |
| B′ | 3/16 | 4/128 | 0·113 | 6/16 | 8/128 | 0·195 | 8/16 | 10/128 | 0·344 |
| C′ | 6/16 | 7/128 | 0·105 | 6/16 | 9/128 | 0·188 | 7/16 | 9/128 | 0·336 |
| Year 2 | 14/48 | 16/384 | 0·103 | 19/48 | 25/384 | 0·190 | 26/48 | 38/384 | 0·349 |
| Total | 30/101 | 39/808 | 0·121 | 40/101 | 54/808 | 0·205 | 60/102 | 78/816 | 0·351 |

Frequency of positive clusters expressed as number of clusters containing one or more positive samples in the 8/number of clusters examined.

Frequency of positive diets expressed as number of positive diet founds ($\geqslant 1$ μg/kg)/total number of diets analysed.

Mean aflatoxin contamination expressed as μg/kg wet diet including all negative samples.

TABLE II.—*Statistics of the Frequencies of Aflatoxin-positive Clusters and of Individual Diet Samples*

| Statistic | Degrees of freedom | $\chi^2$ using +ve clusters | $\chi^2$ using +ve samples |
|---|---|---|---|
| Between altitude sub-areas | 2 | 18·21*** | 14·13*** |
| Between administrative divisions | 3 | 6·06 | 6·18 |
| Between seasons | 6 | 3·98 | 5·61 |
| Between years | 1 | 0·36 | 0·09 |
| Between samples within clusters [1] | 7 | — | 6·83 |
| Within clusters from the binomial [2] | 3 | — | 1·95 |

[1] Starting with the actual cluster centre the set of 8 samples were sequentially marked stroke 1 to stroke 8. This statistic shows no bias for the first or any other sample within a cluster to be contaminated.
[2] Using an overall positivity of 7% (171/2432) the expectations of multiple contaminated samples within a cluster have been calculated from the binomial expansion.
*** Significant at $P < 0\cdot001$ level.

TABLE III.—*Statistics (" t " tests) of the Mean Levels of Dietary Aflatoxin*

| Mean values compared* | | *t* value | Probability |
|---|---|---|---|
| High area year 1 | *v* High area year 2 | 0·78 | >0·10 |
| Middle area year 1 | *v* Middle area year 2 | 0·39 | >0·10 |
| Low area year 1 | *v* Low area year 2 | 0·04 | >0·10 |
| Year 1 | *v* Year 2 | 0·49 | >0·10 |
| Season A | *v* Season A′ | | |
| (widest difference in replicate seasons) | | 0·68 | >0·10 |
| Season A | *v* Season D | | |
| (widest difference in seasons within years) | | 0·62 | >0·10 |
| Season A | *v* Season C′ | | |
| (widest difference between any two seasons) | | 0·65 | >0·10 |
| High area | *v* Middle area | 1·91 | $0\cdot10 > P > 0\cdot05$ |
| High area | *v* Low area | 3·71*** | $P < 0\cdot001$ |
| Middle area | *v* Low area | 2·12* | $0\cdot05 > P > 0\cdot02$ |

* Means including all negative results.

Table III where the mean contamination levels have been evaluated for significance.

The overall result is that the only significant differences occur between altitude areas and this is true of both frequency of contamination and mean contamination level.

In Table IV the principal results obtained in the survey have been summarized and in Fig. 2 the calculated exposure data for males and females in the 3 areas is plotted against the liver cancer data available to date. In arriving at the exposure data a daily intake of 2 kg wet diet by 70 kg adults and of 2 litres of native beer by the males only have been assumed; these figures have been derived from the data collected during this study and the work of Bohdal, Gibbs and Simmons (1969). In Fig. 2 the calculated regression line, $y = 19{\cdot}06 \log_{10} x - 10{\cdot}16$ has been drawn; the correlation coefficient for this line is 0·87 for 4 degrees of freedom ($0{\cdot}05 > P > 0{\cdot}02$).

### DISCUSSION

This study was originally undertaken to establish an aflatoxin level and liver cancer incidence relating to the whole of Murang'a district, which was to be later compared with a similar study carried out in an area of much higher liver cancer incidence. As stated earlier, the study was used to test the feasibility and methodology in relation to both the collection of samples and the analysis of mixed whole diets. The fact that we have been able to sub-divide the data and establish a significant association encourages the extension of this type of study.

Although the results reported here

FIG. 2.—Correlation of liver cancer with aflatoxin ingestion. $y = 19 \cdot 06 \log_{10} x - 10 \cdot 16$, $r = 0 \cdot 87$, $0 \cdot 05 > P_4 > 0 \cdot 02, t\sigma_r = \pm 7 \cdot 55$.

TABLE IV.—*Summary of the Principal Results*

| Altitude Sub-area | High M | High F | Middle M | Middle F | Low M | Low F | Σ = Murang'a M | Σ = Murang'a F |
|---|---|---|---|---|---|---|---|---|
| Total population *ex* 1962 census | 18394 | 20244 | 75138 | 86467 | 68808 | 75803 | 162340 | 182514 |
| Population ⩾ 16 years old (1962 census) | 8027 | 10885 | 30105 | 45693 | 30949 | 41375 | 69081 | 97953 |
| Frequency of aflatoxin-contaminated diets | 39/808 | | 54/808 | | 78/816 | | 171/2432 | |
| Mean Level (μg/kg) | 0·121 | | 0·205 | | 0·351 | | 0·226 | |
| Frequency of aflatoxin-contaminated beers | 3/101 | | 4/101 | | 9/102 | | 16/304 | |
| Mean Level (μg/l) | 0·050 | | 0·069 | | 0·167 | | 0·095 | |
| Mean aflatoxin ingested* ng/kg body weight/day | 4·88 | 3·46 | 7·84 | 5·86 | 14·81 | 10·03 | 9·18 | 6·46 |
| Primary liver cancer cases ⩾16 years old 1967–1970 | 1 | 0 | 13 | 6 | 16 | 9 | 30 | 15 |
| Incidence Rates per $10^5$ per annum | 3·11 | 0·00 | 10·80 | 3·28 | 12·92 | 5·44 | 10·86 | 3·83 |

* Calculated assuming 2 kg intake food and 2 l intake beer (men only) per day and 70 kg average adult body weight.

show a statistically significant association between aflatoxin ingested levels and the liver cancer cases we were able to allocate to the altitude areas within Murang'a, this requires qualification before the aetiological significance is considered. It should be appreciated that a few undetected cases of liver cancer in the high altitude area would completely change the statistical significance of the graph we have derived and that the cancer data have been obtained under far from ideal conditions. The possible anomaly of attempting to relate current cancer to current exposure is ignored and we are aware of the necessity to continue registration to be able to evaluate this exposure study more completely.

Linsell (1967), using biopsy material, reported that the Kamba tribe of Kenya had a frequency of liver cancer approximately twice that of the Kikuyu. The low altitude area of this study is similar to the rolling plains of the adjoining Kamba country and the dietary habits of the low-area Kikuyu resemble those of their Kamba neighbours. The present data accords with this. The lack of a statistically significant seasonal effect was unexpected, considering that aflatoxin contamination is primarily a storage problem, but it could confirm that housewife screening of staples in Murang'a may invalidate analysis of market samples as a measure of ingested aflatoxin. The consistently low contamination level of the local beers (16 positive out of 304 tested—all in the range 1–2·5 μg/l) was also surprising since rejected maize is often used as a carbohydrate source in the beer fermentation. It is possible that aflatoxin is partially destroyed during this type of fermentation and work is in hand to test this.

It is difficult to comment on the levels of aflatoxin in dietary samples and their possible relationship to human liver cancer as the susceptibility to aflatoxin varies so widely between, and even within, species (Butler, 1969). If one assumes that the role of aflatoxin is one of chronic ingestion then the mean dietary level, including all the negatives, will be 0·23 μg/kg. Aflatoxin has been shown to be a single-dose carcinogen operating a year after exposure in rats (Carnaghan, 1967) and therefore it could operate in man as a single-dose on a background of tolerance of small doses and the level for Murang'a would be 3·2 μg/kg, the mean of positive samples only. This is still considered low when compared with the levels that could be expected if groundnuts were a regular inclusion in the Murang'a diet.

A survey of the dietary components included in the positive aflatoxin diets as opposed to the negative diets revealed that maize, millet, sorghum, pigeon peas, cabbage and yams appear to be included more frequently in the positive diets. The cabbage can probably be ignored as it is normally included as a fresh vegetable but the other staples are all dietary constituents in which aflatoxin contamination could be expected since they are often stored under conditions which are far from ideal.

Although more commonly associated with groundnuts, aflatoxin has been demonstrated in a wide range of dietary components (Loosmore *et al.*, 1964). However, the aflatoxin level is not usually so high with natural contamination of these foodstuffs and groundnuts appear to be a substrate of choice for extensive natural aflatoxin production by *A. flavus*. Based on nutritional data available for Murang'a district (Bohdal *et al.*, 1969), maize, the principal suspect dietary staple, would normally constitute about 40% of the dry weight of average diets. The aflatoxin contamination level of the positive diets varied from 1 μg/kg to 21 μg/kg and these levels would be equivalent to contamination levels of 7–160 μg/kg in the original maize at 10% moisture; this appears to be a reasonable range for natural contamination levels in maize (Golumbic cited by Griffiths, 1966; Peers, unpublished data; Alpert *et al.*, 1971).

A possible bias associated with the high proportion of liver cancer cases from the low area may be the site of the main district hospital within this area. We have 12 potential "cells" in the study area: 4 administrative divisions which run east to west by 3 altitude areas which run north to south. Preliminary allocation of the 45 "usable" liver cancer cases to these cells, together with a study of the hospitals from which they registered, did not suggest that there was a marked distance bias and tended to confirm that most cases are likely to reach medical care. We have further tested this possible bias using (i) the combined tuberculosis records for Murang'a district for the years 1964 and 1967; and (ii) the total cancers other than liver recorded during 1967–1970.

TABLE V.—*Comparison of the Distribution of Liver Cancer, Cancers other than Liver and Tuberculosis within Murang'a District*

| Area | | Liver* cancer | Other cancers | Tuberculosis |
|---|---|---|---|---|
| High | observed | 1 | 19 | 93 |
| | expected | 5·1 | 23·8 | 91·6 |
| Middle | observed | 19 | 94 | 375 |
| | expected | 20·4 | 99·4 | 383·3 |
| Low | observed | 25 | 99 | 350 |
| | expected | 19·5 | 88·9 | 343·0 |
| Cases not allocated or used | | 3 | 14 | 87 |
| $\chi^2$ for 2 d.f. | | 4·95 | 2·39 | 0·34 |

* Expected values for liver cancer calculated from 1962 adult populations; for the other cancers and TB the total population data was used.

As can be seen from Table V, the distribution of liver cancer is tending towards significance ($0{\cdot}10 > P_2 > 0{\cdot}05$) and although a similar trend is found with the cancers other than liver the effect is much less significant ($0{\cdot}50 > P_2 > 0{\cdot}30$). The tuberculosis data, where we have much larger numbers of cases shows no such trend ($0{\cdot}90 > P_2 > 0{\cdot}80$) and this data would tend to support the fact that chronic disease cases are not deterred from attending hospital by increasing distance from the medical facilities.

Newberne (1965) published a 4-level regression line ($y = 49{\cdot}75 \log_{10} x - 74{\cdot}61$) for aflatoxin content of diet ($x$) and percentage of liver cancer induction in rats ($y$) read over approximately a one-year period. His groups were of 9–15 rats at the various levels and his lowest disease incidence was about 1%, whereas in the human situation we are attempting to relate aflatoxin exposures to incidence rates of the order of 0·005%. Hence it is most unlikely that such experimental data would cover the lower reaches of a correlation line or curve relevant to the human situation. However, it is of interest that our correlation line takes the same algebraic form as that determined experimentally for rats and for rainbow trout (Sinnhuber *et al.*, 1968).

Correlation does not necessarily prove causation and the above data at best only demonstrate an association between aflatoxin ingestion levels and liver cancer in the Murang'a district. Korobkin and Williams (1968) have found a significant correlation between the homes of liver cancer patients and the distribution of groundnut cultivation in the West Nile District of Uganda. Alpert *et al.* (1971), working on stored foods, conclude that aflatoxin exposure may account for the varying incidence of hepatoma within Uganda. Keen and Martin (1971) have demonstrated that the pattern of occurrence of primary liver cancer in Swaziland is paralleled by the availability of aflatoxin-contaminated groundnuts as determined by home-stored and market samples of groundnuts. Shank and his colleagues (personal communication, 1971) have obtained results in Thailand consistent with the hypothesis that aflatoxin exposure and hepatocellular carcinoma may be related. This study presents further evidence of a more direct kind that aflatoxin may be involved in the aetiology of primary liver cancer.

It is stressed that the Murang'a findings cover only a small range of the possible association and that further studies must be carried out in areas with a higher rate of liver cancer or higher dietary levels of aflatoxin in order to test the strength and consistency of this association. This can be effected by similar dietary studies or possibly by surveys for urinary metabolites of aflatoxin in human populations; this latter possibility has become more feasible as an index of aflatoxin exposure since the demonstration of conjugated aflatoxin in urine (Bassir and Osiyemi, 1967, 1969; Dalezios, Wogan and Weinreb, 1971).

The possibility of removing the potential carcinogen from the environment of a selected population is certainly difficult but the progressive urbanization which is occurring in developing countries may afford an opportunity of determining whether the liver cancer risk is decreasing with urbanization and the changes in food habits and sources of dietary staples.

The considerable assistance offered by the many members of the Ministry of Health of Kenya and the Administrative Officers of the Murang'a District is acknowledged. We are particularly grateful to our collection team, Mr Samuel Mwangi and Mr Peter Mbugwa of Murang'a District for their diligence and hard work, often under trying climatic conditions. We would also like to thank Mr Svend Christensen of the W.H.O. Epidemiology Centre, Nairobi, and Dr N. E. Day of I.A.R.C., Lyon, for statistical help; Dr N. R. Jones, Dr B. D. Jones, and Dr A. J. Feuell of T.P.I. for helpful comments during the preparation of this manuscript, and the Directors of T.P.I. and I.A.R.C. for permission to publish this paper.

### REFERENCES

ALPERT, M. E., HUTT, M. S. R., WOGAN, G. N. & DAVIDSON, C. S. (1971) Association between Aflatoxin Content of Food and Hepatoma Frequency in Uganda. *Cancer, N.Y.*, **28**, 253.

ANDRELLOS, P. J. & REID, G. R. (1964) Confirmatory Tests for Aflatoxin $B_1$. *J. Ass. Off. agric. Chem.*, **47**, 801.

BASSIR, O. & OSIYEMI, F. (1967) Biliary Excretion of Aflatoxin in the Rat after a Single Dose. *Nature, Lond.*, **215**, 882.

BASSIR, O. & OSIYEMI, F. (1969) Urinary Excretion of Aflatoxin after a Single Dose. *W. Afr. J. Biol. appl. Chem.*, **12**, 19.

BOHDAL, M., GIBBS, N. E. & SIMMONS, W. K. (1969) *Nutrition Survey and Campaign against Malnutrition in Kenya, 1964–68.* Report to Ministry of Health, Kenya. WHO/FAO/UNICEF Project.

LE BRETON, E., FRAYSSINET, C. & BOY, J. (1962) Sur l'apparition d'hepatomes " spontanés " chez le Rat Wistar. Role de la Toxine de l'Aspergillus Flavus. Interet en Pathologie Humaine et Cancerologie Experimentale. *C.r. Acad. Sci. (Paris)*, **225**, 784.

BUTLER, W. H. (1969) In *Aflatoxin: Scientific Background, Control and Implications.* Ed. L. A. Goldblatt. New York: Academic Press, p. 223.

CARNAGHAN, R. B. A. (1967) Hepatic Tumours and other Chronic Liver Changes in Rats following a Single Oral Administration of Aflatoxin. *Br. J. Cancer*, **21** 811.

CHRISTENSEN, C. M. (1957) Deterioration of Stored Grains by Fungi. *Botan. Rev.*, **23**, 108.

COOMES, T. J., CROWTHER, P. C., FRANCIS, B. J. & STEVENS, L. (1965) The Detection and Estimation of Aflatoxins in Groundnuts and Groundnut Materials. *Analyst.*, **90**, 492.

CROWTHER, P. C. (1968) Metabolite of *Macrophomina phaseoli* (Maubl) Ashby with TLC Behaviour Similar to that of Aflatoxin B. *Analyst*, **93**, 623.

CUCULLU, A. F., LEE, L. S., MAYNE, R. Y. & GOLDBLATT, L. A. (1966) Determination of Aflatoxins in Individual Peanuts and Peanut Sections. *J. Am. Oil Chem. Soc.*, **43**, 89.

DALEZIOS, J., WOGAN, G. N. & WEINREB, S. M. (1971) Aflatoxin $P_1$—a New Aflatoxin Metabolite in Monkeys. *Science, N.Y.*, **171**, 584.

DAVIES, J. N. P. & OWOR, R. (1960) The Diagnosis of Primary Carcinoma of the Liver. *E. Afr. med. J.*, **37**, 249.

EPPLEY, R. M. (1968) Screening Method for Zearalenone, Aflatoxin and Ochratoxin. *J. Ass. Off. agric. Chem.*, **51**, 74.

GRIFFITHS, D. L. (1966) *Review of U.S.D.A. Research on Mycotoxins. United Kingdom Scientific Mission (N. America).* Rep. No. 66/24.

HARTLEY, R. D., NESBITT, B. F. & O'KELLY, J. (1963) Toxic Metabolites of *A. flavus*. *Nature, Lond.*, **198**, 105.

KEEN, P. & MARTIN, P. (1971) Is Aflatoxin Carcinogenic in Man? The Evidence in Swaziland. *Trop. geogr. Med.*, **23**, 44.

*Kenya Population Census, 1962* (1964). Vol. 1.

KOROBKIN, M. & WILLIAMS, E. H. (1968) Hepatoma and Groundnuts in the West Nile District of Uganda. *Yale J. Biol. Med.*, **41**, 69.

LINSELL, C. A. (1967) Cancer Incidence in Kenya, 1957–1963. *Br. J. Cancer*, **21**, 465.

LOOSMORE, R. M., ALLCROFT, R., TUTTON, E. A. & CARNAGHAN, R. B. A. (1964) The Presence of Aflatoxin in a Sample of Cottonseed Cake. *Vet. Rec.*, **76**, 64.

NABNEY, J. & NESBITT, B. F. (1964) Determination of the Aflatoxins. *Nature, Lond.*, **203**, 862.

NEWBERNE, P. M. (1965) In *Mycotoxins in Foodstuffs.* Ed. G. N. Wogan. Cambridge, Mass.: M.I.T. Press, p. 190.

NEWBERNE, P. M. & BUTLER, W. H. (1969) Acute and Chronic Effects of Aflatoxin on the Liver of Domestic and Laboratory Animals—a Review. *Cancer Res.*, **29**, 236.

O'CONOR, G. T., TATARINOV, Y. S., ABELEV, G. I. & URIEL, J. (1970) Collaborative Study for the Evaluation of a Serological Test for Primary Liver Cancer. *Cancer, N.Y.*, **25**, 1091.

OETTLE, A. G. (1965) The Etiology of Liver Carcinoma in Africa with an Outline of the Mycotoxin Hypothesis. *S. Afr. med. J.*, **39**, 817.

POHLAND, A. E., YIN, L. & DANTZMAN, J. G. (1970) Rapid Chemical Confirmatory Method for Aflatoxin $B_1$. *J. Ass. Off. agric. Chem.*, **53**, 101.

PONS, W. A. (1968) Fluorodensitometric Measurement of Aflatoxins on TLC Plates. *J. Ass. Off. agric. Chem.*, **51**, 913.

PONS, W. A., ROBERTSON, J. A. & GOLDBLATT, L. A. (1966) Objective Fluorometric Measurement of Aflatoxins on TLC Plates. *J. Am. Oil Chem. Soc.*, **43**, 665.

PONS, W. A. & GOLDBLATT, L. A. (1969) In *Aflatoxin: Scientific Background, Control and Implications.* Ed. L. A. Goldblatt. New York: Academic Press, p. 77.

SINNHUBER, R. O., LEE, D. J., WALES, J. H. & AYRES, J. L. (1968) Dietary Factors and Hepatoma in Rainbow Trout. II. Co-carcinogenesis by Cyclopropenoid Fatty Acids and the Effect of Gossypol and Altered Lipids on Aflatoxin Induced Liver Cancer. *J. natn. Cancer Inst.*, **41**, 1293.

STOLOFF, L., BECKWITH, A. C. & CUSHMAC, M. E. (1968) TLC Spotting Solvent for Aflatoxins. *J. Ass. Off. agric. Chem.*, **51**, 65.

WILEY, M., WAISS, A. C. & BENNETT, N. (1969) Reaction of Aflatoxin $B_1$ with Acetic Acid–Thionyl Chloride. *J. Ass. Off. agric. Chem.*, **52**, 75.